# Decreased Platelet Specific Receptor Expression of P-Selectin and GPIIb/IIIa Predict Future Non-Surgical Bleeding in Patients after Left Ventricular Assist Device Implantation

**DOI:** 10.3390/ijms231810252

**Published:** 2022-09-06

**Authors:** Kristin Klaeske, Anna L. Meyer, Diyar Saeed, Sandra Eifert, Khalil Jawad, Franz Sieg, Josephina Haunschild, Michael A. Borger, Maja-Theresa Dieterlen

**Affiliations:** 1 Department of Cardiac Surgery, Heart Center Leipzig, University of Leipzig, 04289 Leipzig, Germany; 2 Department of Cardiac Surgery, University Hospital Heidelberg, 69120 Heidelberg, Germany

**Keywords:** mechanical circulatory support, heart failure, non-surgical bleeding, platelet dysfunction, non-physiological shear stress

## Abstract

Non-surgical bleeding (NSB) is one of the major clinical complications in patients under continuous-flow left ventricular assist device (LVAD) support. The increased shear stress leads to an altered platelet receptor composition. Whether these changes increase the risk for NSB is unclear. Thus, we compared the platelet receptor composition of patients with (bleeder group, *n* = 18) and without NSB (non-bleeder group, *n* = 18) prior to LVAD implantation. Blood samples were obtained prior to LVAD implantation and after bleeding complications in the post-implant period. Platelet receptor expression of GPIbα, GPIIb/IIIa, P-selectin and CD63 as well as intra-platelet oxidative stress levels were quantified by flow cytometry. Bleeders and non-bleeders were comparable regarding clinical characteristics, von Willebrand factor diagnostics and the aggregation capacity before and after LVAD implantation (*p* > 0.05). LVAD patients in the bleeder group suffered from gastrointestinal bleeding (33%; *n* = 6), epistaxis (22%; *n* = 4), hematuria or hematoma (17%; *n* = 3, respectively) and cerebral bleeding (11%; *n* = 2). Prior to LVAD implantation, a restricted surface expression of the platelet receptors P-selectin and GPIIb/IIIa was observed in the bleeder group (P-selectin: 7.2 ± 2.6%; GPIIb/IIIa: 26,900 ± 13,608 U) compared to non-bleeders (P-selectin: 12.4 ± 8.1%, *p* = 0.02; GPIIb/IIIa: 36,259 ± 9914 U; *p* = 0.02). We hypothesized that the reduced platelet receptor expression of P-selectin and GPIIb/IIIa prior to LVAD implantation may be linked to LVAD-related NSB.

## 1. Introduction

Left ventricular assist device (LVAD) implantation is an effective therapeutic option for patients with end-stage heart failure [1,2]. Despite the clinical outcomes of prolonged survival and improved quality of life, LVAD usage is associated with various LVAD-related adverse events such as infections, pump failure, thrombosis or non-surgical bleedings (NSB) [2]. The incidence of NSB is defined as intracranial hemorrhage events requiring two units of packed red blood cells, or death from bleeding occurring > 7 days after LVAD implantation, and was reported in 41% of LVAD patients with 0.48 events per patient year [3]. NSB contributes significantly to the 1-year survival rate in patients with LVAD support [4]. The pathophysiological mechanism underlying NSB remains unclear [1,5].

In LVAD patients, the permanent exposure of blood components to device-induced non-physiological shear stress disrupts the hemostasis [4]. The documented changes in blood components during LVAD support are the activation of platelets, changes in platelet receptors [6] and the reduction in large multimers of the von Willebrand factor (vWF) leading to the development of the acquired von Willebrand syndrome (aVWS), which is associated with bleeding [7,8]. Because aVWS occurs in practically all LVAD patients, but not all LVAD patients develop bleeding complications, changes in platelets seem to be relevant to developing NSB [8].

Elevated oxidative stress biomarkers, platelet receptor shedding of glycoprotein (GP) Ibα, GPVI and an activation of GPIIb/IIIa were reported in patients with post-implant bleeding complications [6,9,10,11,12,13,14]. Therefore, the loss of functional surface receptors results in a defective platelet function and may contribute to an increased bleeding risk in LVAD patients. It is important, to understand the central role of platelet dysfunction in the pathogenesis of NSB to possibly provide a tool for bleeding risk stratification in LVAD patients. In this study, we investigated whether the platelet receptor composition of patients with future bleeding complications differs from patients without bleeding complications prior to LVAD implantation.

## 2. Results

### 2.1. Patient Characteristics

Both study groups were matched for age at LVAD implantation (bleeders: 62.2 ± 10.8 years, non-bleeders: 59.6 ± 7.8) and male sex (bleeders: 83%, non-bleeders: 72%). LVAD patients with and without NSB were comparable regarding body mass index, etiology, blood type, left ventricular ejection fraction, New York Heart Association (NYHA) class, comorbidities, smoking and alcohol status prior to LVAD implantation as well as regarding the anticoagulation therapy after LVAD implantation (Table 1). All patients received phenprocoumon and most of the patients were additionally anticoagulated with acetylsalicylic acid or clopidogrel. LVAD parameters showed that the pump power was significantly reduced in the non-bleeder group (*p* = 0.02), while pump speed and pump flow were comparable in both groups (Table 1).

Thromboembolic events prior to and at 1-year post-implant did not differ between bleeders and non-bleeders (Table 2).

**Figure 1:**
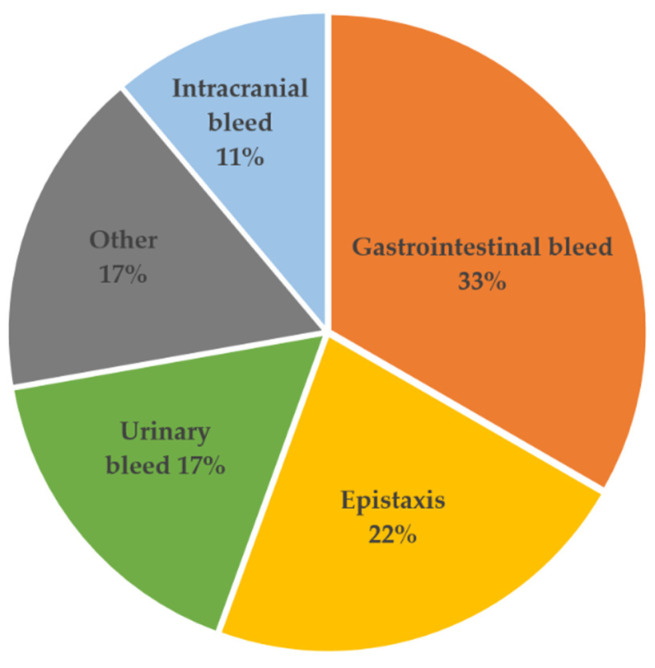
Percentage of types of bleeding after left ventricular assist device implantation in this study.

In Table 3, we recorded and characterized the first bleeding event within 1 year of LVAD implantation. After surgery the most common source of bleeding was gastrointestinal (33%), followed by epistaxis (22%), urinary tract-related causes and hematoma (17%, respectively) as well as intracranial bleeding events (Figure 1). Major bleeding events were identified in 61% of patients, and 39% had a minor bleeding event. The incidence of NSB occurred on average 70 ± 79 days after LVAD implantation (Table 3).

The hemoglobin content and erythrocyte count were significantly decreased in bleeders compared to non-bleeders prior to LVAD implantation (Table 4). The hematocrit, INR, platelet count, activated partial thromboplastin time, bilirubin and lactate dehydrogenase were comparable between bleeders and non-bleeders prior to LVAD implantation. The hematocrit and hemoglobin content were out of reference range in 86% and 89%, respectively, of the patients prior to LVAD implantation.

Three months after LVAD implantation, vWF diagnostics including the quantification of vWF antigen, vWF activity, factor VIII procoagulant and vWF collagen-binding activity were comparable between bleeders and non-bleeders (Table 5).

### 2.2. Analysis of Platelet Receptor Expression and Platelet Aggregation Measurements

**Figure 2:**
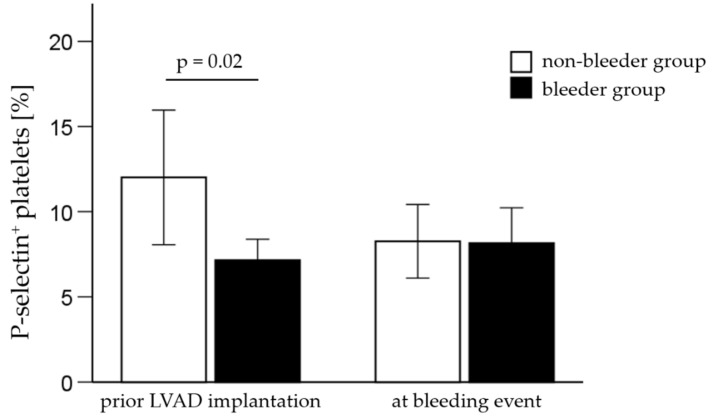
The percentage of P-selectin-positive platelets in patients with (bleeder) and without bleeding complications (non-bleeder) prior to LVAD implantation and at the timepoint of the first bleeding event. All values are expressed as mean ± standard error. *p* ≤ 0.05 is considered statistically significant.

Flow cytometry results, including the percentage and mean fluorescence intensity (MFI) of platelet receptor expression are summarized in Table 6 and Table 7. The percentage of platelet receptor P-selectin was significantly reduced (*p* = 0.02) in the bleeder group compared to the non-bleeder group on platelets prior to LVAD support (Figure 2).

**Figure 3:**
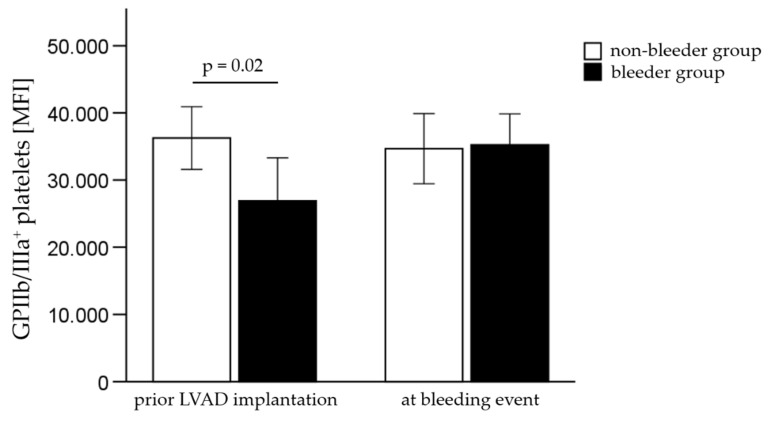
The relative quantity of GPIIb/IIIa molecules on platelets in patients with (bleeder) and without bleeding complications (non-bleeder) prior to LVAD implantation and at the timepoint of the first bleeding event. All values are expressed as mean ± standard error. *p* ≤ 0.05 is considered statistically significant.

Furthermore, the surface expression level of GPIIb/IIIa on platelets, measured by the MFI, was significantly decreased (*p* = 0.02) in bleeders compared to non-bleeders prior LVAD implantation (Figure 3, Table 6).

**Figure 4:**
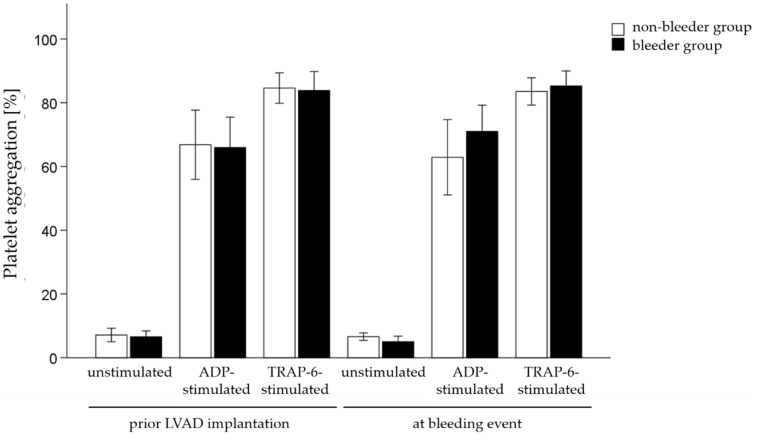
Platelet aggregation of unstimulated platelets and platelets stimulated with ADP or thrombin receptor-activating peptide (TRAP-6) in patients with (bleeder) and without bleeding complications (non-bleeder). All values are expressed as mean ± standard error. *p* ≤ 0.05 is considered statistically significant.

The platelet function was measured by aggregometry testing before and after LVAD implantation. Results showed that neither the percentage of unstimulated platelets, nor ADP-stimulated platelets and TRAP6-activated platelets changed in patients with NSB and those patients who remained free of bleeding complications (Figure 4).

### 2.3. Analysis of Intra-Platelet Oxidative Stress Level

There were no significant differences in the detection of oxidative stress or the measurement of mitochondrial mass between the non-bleeder and the bleeder group before and after LVAD implantation (Table 6 and Table 7).

### 2.4. Serum Analysis of VEGF Expression and Soluble P-Selectin

The quantification of circulating VEGF prior to LVAD implantation and after the first bleeding event revealed that VEGF levels were comparable in patients prior to LVAD implantation (bleeders: 586.1 ± 60.5 pg/mL, non-bleeders: 794.8 ± 203.1 pg/mL, *p* = 0.33) and at the time point of the bleeding event (bleeders: 706.1 ± 143.3 pg/mL, non-bleeders: 563.3 ± 92.4 pg/mL, *p* = 0.41). When both study groups were compared, the serum concentration of soluble P-selectin did not differ significantly in patients pre-implant (bleeders: 118.6 ± 39.5 pg/mL, non-bleeders: 124.8 ± 30.9 pg/mL, *p* = 0.60) and post-implant when NSB occurred (bleeders: 121.1 ± 41.7 pg/mL, non-bleeders: 119.0 ± 31.2 pg/mL, *p* = 0.86).

## 3. Discussion

In this study, we found that patients awaiting LVAD implantation who later suffered from NSB during LVAD support had a reduced surface expression of platelet receptors P-selectin and GPIIb/IIIa. No significant differences were observed in the expression of the platelet receptors GPIbα and CD63, platelet aggregation capacity or intra-platelet oxidative stress levels between patients with and without NSB. We suggest that routine measurements of P-selectin and GPIIb/IIIa prior to LVAD implantation could potentially identify patients at risk for LVAD-related NSB.

The balance of bleeding and thrombosis is a central component in the therapeutic management of patients supported by LVAD. In this context, impaired hemocompatibility results from increased shear stress, low pulsatility and the complex interaction of patient’s blood components with the artificial interface of LVAD pumps, thereby triggering coagulation abnormalities [8,15,16,17]. Many studies have focused on the clinical characteristics associated with bleeding complications after LVAD implantation, identifying age, history of prior bleeding, low platelet count and comorbidities such as renal insufficiency or hypertension as risk factors for post-LVAD bleeding complications [1,5,18,19,20,21]. In our study, age, pre-implant bleeding rate, platelet count and recorded comorbidities were comparable in patients with and without NSB. In addition, the timing of the first bleeding event was not limited to a distinct period after LVAD implantation. This is consistent with other studies, in which bleeding events range from the early postoperative phase (≤3 month) until 2 years after LVAD implantation [2,21]. Furthermore, the postoperative anticoagulation regime was comparable between the bleeder and the non-bleeder group. However, the influence of pre-operative (pre-LVAD) anticoagulation on platelets could not be assessed in the setting of this study.

In the past, various hemocompatibility parameters (e.g., vWF diagnostic, generation of reactive oxygen species, platelet aggregation) have been investigated to identify a reliable biomarker for bleeding risk stratification in LVAD patients [4,8,17,22]. A combined examination for angiodysplasia and aVWS seemed to be able to explain the occurrence of NSB [23,24,25,26,27]. Today, it is already known that the majority of LVAD patients develop an aVWS by the loss of high molecular multimers; however, the analyzed vWF profiles are not suitable to discriminate between patients with and without bleeding complications [6,8,24]. In our study, angiogenesis measured by VEGF expression and the vWF profile was comparable in patients with and without NSB.

Since platelets play a pivotal role in hemostasis, the device-induced platelet dysfunction may contribute to severe bleeding complications under LVAD support. Chen and colleagues conducted a series of in vitro experiments to investigate the influence of high non-physiological shear stress (NPSS) on the structural integrity of platelet receptors in blood contacting medical devices. They found that NPSS caused a paradoxical phenomenon [10]. On the one hand, shear stress-induced platelet activation increases the risk of thrombosis. On the other hand, blood exposed to NPSS contributes to platelet receptor shedding and strengthens the propensity of bleeding. Chen et al. demonstrated more activated platelets by the increased number of GPIIb/IIIa-expressing platelets and P-selectin expression in sheared blood samples compared to normal blood. However, they showed the decreased mean fluorescence of GPIIb/IIIa on the platelet surface and the enhanced GPIIb/IIIa concentration in human plasma advocated for platelet receptor shedding [10,11,12]. The reduction in functional platelet receptor GPIIb/IIIa lowers the adhesion capacity of platelets for fibrinogen and vWF binding, and therefore enhances the risk for bleeding [12]. In the recent study, we detected a decrease in the relative quantity of GPIIb/IIIa molecules on platelets in bleeders compared to non-bleeders prior to LVAD implantation.

As well as the modified platelet surface expression, intrinsic and extrinsic signaling pathways seemed to change in patients after LVAD implantation. In our study, a secretion defect of α-granules in platelets was suspected in patients with bleeding complications because of a pre-implant reduced percentage of P-selectin-positive platelets. Furthermore, the levels of soluble P-selectin were similar in serum samples of the bleeder and the non-bleeder groups at both time points. The findings suggest a platelet dysfunction due to the reduction in P-selectin-positive platelets originating from the platelets itself, even before LVAD implantation, which is augmented by the LVAD-induced abnormally NPSS. Previous studies reported an association between a platelet-secretion defect resulting in hypoaggregability of platelets and an impaired coagulation system in patients with temporary venous extracorporeal membrane oxygenation support and after LVAD implantation [28,29]. The resulting platelet dysfunction and the aVWS exacerbates the bleeding risk in those patients.

As well as the mechanisms of hemostasis and thrombosis, platelets recognize and respond to a variety of pathogens, leading to platelet activation through their receptors and mediation of an immune response. For example, P-selectin-positive platelets support the recruitment of circulating immune cells during infection. [30]. However, the role of platelets in the detection and regulation of infection before and after LVAD implantation should be addressed in future study.

Moreover, our results showed no differences in ROS generation and mitochondrial mass between patients with and without NSB. It was previously shown that end-stage heart failure patients do not have higher grades of oxidative stress after LVAD implantation [31]. However, different types of blood cells showed a higher ROS generation in bleeders than in non-bleeders [9].

In this study, there were no significant differences in functional platelet testing. A comparable clotting time in patients with and without bleeding complications might be due to the optimal setting of anticoagulation treatments in the follow-up period. All patients were regularly monitored by aggregometry, and INR adjustment was performed, if necessary.

Finally, the detailed pathophysiological mechanism leading to bleeding complications in LVAD patients has not been thoroughly identified and depends on several patient-specific factors and presumably genetic factors in addition to shear-stress-induced changes in the coagulation system. This underlines the importance of an early combined measurement of a set of platelet receptors, clinical markers and scoring systems to evaluate the bleeding risk in patients awaiting LVAD implantation.

The analysis of the preexisting decreased expression of P-selectin and GPIIb/IIIa in HF patients before LVAD implantation potentially contributes to a better risk assessment of bleeding as a consequence of impaired platelet function. Consequently, this could contribute to an individual patient-specific adjustment of the anticoagulation regimen after LVAD implantation, which in turn might reduce bleeding complications in these patients. The platelet receptor composition is inevitable for the physiological platelet function and should be considered in patients undergoing LVAD implantation because of bleeding risk stratification despite normal platelet counts, aVWD and aggregation capacity. An individualized anticoagulation with monotherapy of phenprocoumon or an adjustment of antiplatelet therapy could be possible clinical concepts.

We acknowledge that our observational study is limited by its monocentric, retrospective design and the small sample size. In retrospective studies, the results may be influenced by confounding factors. A prospective study is required to assess the utility of our findings in relation to impaired platelet physiology before LVAD implantation. A larger cohort of study patients with stringent and validated selection criteria could be used to verify our initial hypothesis. Furthermore, the absence of platelet receptor differences after LVAD implantation needs to be explored. Another limitation is that effects of prior medication, concomitant infections such as driveline infections, or comorbidities on platelet function cannot be excluded with our study design, and require further investigation. In particular, prior anticoagulation therapy in patients with prior myocardial infarction and the effect on platelet receptor function after LVAD implantation should be screened in more detail. In addition, there is a risk of alpha error inflation due to statistical testing of different platelet receptors and markers for oxidative stress. Thus, results are exploratory and should be proven in further investigations.

## 4. Materials and Methods

### 4.1. Study Groups and Clinical Characteristics

The study was approved by the Ethics Committee of the Medical Faculty from the University of Leipzig, Germany (ID: 225/17-ek). All patients gave their written informed consent.

A total of 36 end-stage heart failure patients who received LVAD implantation between July 2019 and December 2020 at the Heart Center Leipzig were retrospectively matched. Matching by age and sex was performed for patients with and without NSB (*n* = 18 in each group). NSB was defined as gastrointestinal bleeding (e.g., melena or upper gastrointestinal bleeding according to Forrest classification), hematoma, hematuria epistaxis or intracranial bleeding. Bleeding events were further categorized in major bleeding events, defined as need for blood transfusion, bleeding episodes that required an invasive intervention or involved a critical organ, and minor bleeding events. History of gastrointestinal bleeding and anticoagulation disorder prior LVAD implantation was an exclusion criterion for this study.

Demographic data, clinical characteristic including comorbidities, and pump characteristics were documented. Laboratory values such as hemoglobin, hematocrit, platelet count, international normalized ratio (INR), C-reactive protein level and partial thromboplastin time were recorded at baseline and at the time of the first bleeding event. The vWF diagnostic (vWF antigen, vWF activity, collagen-binding activity and coagulated factor VIII concentration) was performed once at 3 months after LVAD implantation. The anticoagulation regimen was adjusted to phenprocoumon with a target INR of 2.0–2.5 for HM3 patients and INR of 2.5–3.0 for HVAD patients, and patient-specific dosing of clopidogrel or acetylsalicylic acid. In addition, regular hemostaseological diagnostic tests and monitoring of anticoagulation therapy were performed to ensure optimal coagulation conditions.

### 4.2. Blood Sampling

Citrated whole blood and serum was withdrawn prior to as well as 3, 6 and 12 months after LVAD implantation. Sera were centrifuged at 2000× *g* for 10 min, aliquoted and frozen at −20 °C until further analysis.

Flow cytometric analyses and platelet aggregometry testing were started after 60 min incubation at room temperature, and were completed within 2–3 h after blood withdrawal. Platelet-rich plasma (PRP) was prepared from whole blood by centrifugation at 200× *g* for 15 min, and platelet-poor plasma (PPP) by subsequent centrifugation at 1500× *g* for 20 min.

### 4.3. Flow Cytometry

The platelet surface receptors, P-selectin, GPIbα and CD63, and the activated level of platelet receptor GPIIb/IIIa were determined in citrated whole blood samples. A CD41 staining was used to identify platelets. All antibodies were obtained from BioLegend (San Diego, CA, USA). Blood samples were incubated with three different antibody panels for 5 min at 37 °C: panel 1: CD41-PE, CD62P-APC, CD42b-FITC; panel 2: CD41-PE, CD63-PerCP/Cy5.5; panel 3: CD41-PE, CD41/61-PECy7. Afterwards, cells were washed with 4 mL Hanks balanced salt solution followed by centrifugation at 300× *g* for 5 min. For fixation of cells, 500 µL of 1% formalin/phosphate-buffered saline (PBS) was added to each sample before analysis. Flow cytometric analysis was performed using a BD™ LSR II cytometer with *FACS Diva 6.1.3 software* (BD Biosciences, Franklin Lakes, NJ, USA). Standardization of the instrument was performed by weekly measurements of *Cytometer Setup and Tracking Beads* (BD Biosciences). At least 10,000 events were measured per sample and panel.

For analysis of intracellular oxidative stress, we labeled platelets with MitoTracker^®^ Green to detect mitochondrial mass using flow cytometry [32]. PRP was incubated with 50 nM MitoTracker^®^ Green for 15 min at 37 °C. After incubation, cells were centrifuged at 800× *g* for 10 min and washed with 4 mL Tyrode buffer (134 mM NaCl, 12 mM NaHCO_3_, 2.9 mM KCl, 0.34 mM Na_2_HPO_4_, 1 mM MgCl_2_, 10 mM HEPES). The samples were centrifuged at 800× *g* for 10 min and analyzed immediately. An unstained control without MitoTracker^®^ Green served as control.

To evaluate intracellular generation and production of reactive oxygen species, dihydrorhodamine123 (DHR123), a cell-permeable mitochondrial-avid component that oxidized to rhodamine within cells, was measured. PRP samples were incubated with 30 nM DHR123 for 20 min at 37 °C. The reaction was stopped on ice for 10 min. After washing once with 2 mL cold PBS, the samples were centrifuged at 425× *g* for 5 min at 4 °C. An unstained control without DHR123 served as control. Flow cytometric analysis of MitoTracker^®^ Green and DHR123 staining was performed measuring 10,000 events per sample.

### 4.4. Platelet Aggregation Measurements

Platelet aggregation was quantified with PAP4 (MöLab, Langenfeld, Germany) by turbidimetry after stimulation PRP with the platelet agonists adenosine diphosphate and thrombin receptor-activating peptide (TRAP-6). First, 225 µL PRP was incubated at 37 °C with a stirring rate of 1000 rpm. Then, 25 µL adenosine diphosphate or TRAP-6 was added, and aggregation was recorded for 15 min.

### 4.5. Elisa

Serum concentrations of vascular endothelial growth factor (VEGF) and soluble P-selectin were determined using the Human VEGF SimpleStep ELISA Kit (Abcam, Cambridge, UK) and the Human P-selectin Sandwich ELISA Kit (Proteintech, Rosemont, IL, USA), respectively. These assays were performed according to the manufacturer’s instructions. Measurements were recorded with the microplate reader Infinite™ 200 PRO and i-control™ software (both Tecan, Männedorf, Switzerland).

### 4.6. Statistics

Statistical analyses were performed using *SPSS Statistics 28* software (IBM Corp., New York, USA 1989, version 2021). Unless stated otherwise, data are displayed as mean ± standard deviation (SD) or as percentage proportion. The comparison of means for demographic and clinical parameters between the two study groups was executed with the Pearson Chi-Square test or the Yates continuity correction in case of categorical data. Unpaired t-test was used in the case of normal distribution of residuals for two group comparison in case of metric variables. For all analyses, *p*-values < 0.05 were considered as statistically significant. Bonferroni correction for multiple testing was not performed. Statistical significance was evaluated in a 2-sided manner. The two study cohorts were matched by age and sex using the frequency matching strategy.

## 5. Conclusions

In conclusion, this study showed that patients with postoperative bleeding complications had an altered platelet composition prior to LVAD implantation compared to patients without NSB. We suggested that the reduced platelet surface expression of GPIIb/IIIa and P-selectin might be linked to a dysregulated platelet function in future bleeders. Our results of decreased platelet activation may contribute to the understanding of the underlying mechanism of NSB, and, therefore, offer the possibility of bleeding risk stratification by the analysis of P-selectin and GPIIb/IIIa in patients awaiting LVAD implantation. However, this should be validated in future prospective investigations in a larger study cohort.

## Figures and Tables

**Table 1:** Demographic and clinical characteristics of bleeders and the non-bleeders prior to LVAD implantation and LVAD-related parameters.

Parameter	Bleeders	Non-Bleeders	*p*-Value
	(*n* = 18)	(*n* = 18)	
Body mass index (kg/m^2^)	28.0 ± 4.7	27.0 ± 4.9	0.52
Blood type			0.79
A	8 (44%)	9 (50%)	
B	1 (6%)	1 (6%)	
AB	1 (6%)	0 (0%)	
O	8 (44%)	8 (44%)	
Etiology of heart disease			0.18
NICM	6 (33%)	11 (61%)	
ICM	12 (67%)	7 (39%)	
LVEF			
30–40%	1 (6%)	1 (6%)	1.00
<30%	17 (94%)	17 (94%)	1.00
Comorbidities			
hypertension	14 (78%)	18 (100%)	0.1
diabetes mellitus type 2	10 (56%)	7 (39%)	0.51
myocardial infraction	5 (28%)	2 (11%)	0.4
chronic kidney disease	13 (72%)	9 (50%)	0.31
hyperproteinemia	11 (61%)	6 (33%)	0.18
Smoking			0.72
current	3 (17%)	4 (22%)	
former	10 (56%)	7 (39%)	
never	2 (11%)	4 (22%)	
unknown	3 (17%)	3 (17%)	
Alcohol abuse			0.71
current	1 (6%)	1 (6%)	
former	3 (17%)	6 (33%)	
never	10 (56%)	8 (44%)	
unknown	4 (22%)	3 (17%)	
NYHA classification			0.18
class III	8 (44%)	12 (67%)	
class IV	10 (56%)	5 (28%)	
Device strategy			0.51
DT	10 (55%)	8 (45%)	
BTT	5 (28%)	4 (22%)	
BTD/BTC	3 (17%)	6 (33%)	
Implanted LVAD type			1.00
HM 3	15 (83%)	16 (89%)	
HVAD	3 (17%)	2 (11%)	
Pump characteristics			
pump speed )rpm)	4889 ± 1091	4800 ± 855	0.79
pump flow (L/min)	4.1 ± 0.6	3.9 ± 0.4	0.17
pump power (W)	3.9 ± 0.6	3.6 ± 0.3	0.02
Anticoagulation			
phenprocoumon	18 (100%)	18 (100%)	1.00
clopidogrel	1 (6%)	2 (11%)	1.00
acetylsalicylic acid	15 (83%)	16 (89%)	1.00
Hospitalization after LVAD implantation (d)	56 ± 34	48 ± 16	0.38
Re-hospitalization	12 (67%)	12 (67%)	1.00

BTC, bridge to candidacy; BTD, bridge to decision; BTT, bridge to transplantation; DT, destination therapy; HVAD, HeartWare ventricular assist device; HM 3, HeartMate 3; ICM, ischemic cardiomyopathy; LVAD, left ventricular assist device; LVEF, left ventricular ejection fraction; NICM, non-ischemic cardiomyopathy.

**Table 2:** Thromboembolic and hemorrhagic events.

	Bleeders	Non-Bleeders	*p*-Value
	(*n* = 18)	(*n* = 18)	
Thromboembolic event prior LVAD implantation			
thrombosis	8 (44%)	6 (33%)	0.73
ischemic stroke	8 (44%)	6 (33%)	0.73
Hemorrhagic events after LVAD implantation *			-
Gastrointestinal tract	6 (33%)	-	
Vascular (epistaxis)	4 (22%)	-	
Intracranial	2 (11%)	-	
Urinary tract (hematuria)	3 (17%)	-	
other (hematoma)	3 (17%)	-	
Thromboembolic event after LVAD implantation *			
thrombosis	3 (17%)	1 (6%)	0.6
ischemic stroke	0 (0%)	3 (17%)	0.23

* Comprises the first year after LVAD implantation; GI, gastrointestinal; LVAD, left ventricular assist device.

**Table 3:** Detailed information of the first bleeding event after LVAD implantation in the bleeder group.

Patient	Bleeding Site	Symptoms	GI Procedure	Minor/Major	Days Post-Implant
1	GI	Rectal hemorrhage, melena	no	major	93
2	other / intramyocardial	hematoma	no	minor	9
3	GI	melena, Forrest 1b	yes	major	22
4	vascular	epistaxis	no	minor	233
5	vascular	epistaxis	no	minor	55
6	GI	melena	no	major	69
7	GI	melena	no	major	29
8	GI	melena, Forrest 1a	yes	major	128
9	vascular	epistaxis	no	minor	144
10	other/abdominal	retroperitoneal hematoma	no	major	18
11	other/skin	hematoma	no	minor	16
12	intracranial	subdural hematoma	no	major	9
13	intracranial	subarachnoid hemorrhage	no	major	4
14	GI	Forrest 1b	yes	major	26
15	Urinary tract	hematuria	yes	major	112
16	vascular	epistaxis	no	minor	280
17	Urinary tract, GI	hematuria, melena	yes	major	8
18	Urinary tract	hematuria	no	minor	12

GI, gastrointestinal.

**Table 4:** Blood count parameters of bleeders and non-bleeders prior to LVAD implantation and at the timepoint of bleeding event.

Parameter	Bleeders Prior LVAD (*n* = 18)	Non-Bleeders Prior LVAD (*n* = 18)	Bleeders at Bleeding Event (*n* = 18)	*p*-Value Prior LVAD
Erythrocytes (Tpt/L)	3.6 ± 0.6	4.1 ± 0.7	3.1 ± 0.8	0.02
Hematocrit	0.31 ± 0.05	0.34 ± 0.05	0.27 ± 0.07	0.13
Hemoglobin (mmol/L)	6.5 ± 1.0	7.3 ± 1.1	5.6 ± 1.6	0.04
Platelets (Gpt/L)	225 ± 78	253 ± 129	282 ± 60	0.44
INR	1.1 ± 0.2	1.2 ± 0.4	2.1 ± 0.8	0.41
Bilirubin (µmol/L)	15.9 ± 10.8	14.6 ± 9.6	12.6 ± 9.9	0.70
LDH (µmol/(L/s))	3.8 ± 1.1	5.1 ± 3.7	10.9 ± 24.5	0.16
aPTT (sec)	43.3 ± 15.3	47.4 ± 13.6	60.2 ± 29.1	0.41

INR, international normalized ratio; LDH, lactate dehydrogenase; aPTT, activated partial thromboplastin time.

**Table 5:** Von Willebrand and Factor VIII diagnostics in bleeders and non-bleeders 3 months after LVAD implantation.

Parameter.	Bleeders (*n* = 11)	Non-Bleeders (*n* = 10)	*p*-Value
vWF antigen (%)	186.1 ± 52.0	185.7 ± 58.2	0.99
vWF activity (%)	129.1 ± 28.1	138.8 ± 37.4	0.51
vWF CB activity (%)	126.4 ± 40.3	135.1 ± 44.4	0.67
Factor VIII, procoagulant (%)	195.8 ± 64.0	204.2 ± 47.5	0.74

CB, collagen-binding; vWF, von Willebrand factor.

**Table 6:** Flow cytometric analysis of platelet receptor expression and oxidative stress in LVAD patients with and without NSB prior LVAD implantation.

Parameter	Bleeders (*n* = 18)	Non-Bleeders (*n* = 18)	*p*-Value
% P-selectin^+^ platelets	7.2 ± 2.6	12.4 ± 8.1	0.02
% GP1bα^+^ platelets	88.4 ± 25.5	93.4 ± 14.2	0.47
% CD63^+^ platelets	0.57 ± 0.32	3.29 ± 6.14	0.08
% GPIIb/IIIa^+^ platelets	77.8 ± 42.7	94.5 ± 23.6	0.16
% DHR123^+^ platelets	88.4 ± 9.6	84.4 ± 7.7	0.17
% MitoTracker Green^+^ platelets	30.0 ± 25.2	23.5 ± 13.9	0.34
MFI P-selectin^+^ platelets	1227 ± 354	1330 ± 463	0.46
MFI GP1bα^+^ platelets	930 ± 807	674 ± 257	0.21
MFI CD63^+^ platelets	1544 ± 1169	2727 ± 3020	0.14
MFI GPIIb/IIIa^+^ platelets	26,900 ± 13,608	36,259 ± 9913	0.02
MFI DHR123^+^ platelets	742 ± 435	575 ± 285	0.18

CD, cluster of differentiation; DHR123, dihydrorhodamine123; GP, glycoprotein; MFI, mean fluorescence intensity; NSB, non-surgical bleeding.

**Table 7:** Flow cytometric analysis of platelet receptor expression and oxidative stress in LVAD patients with and without NSB at the timepoint of bleeding event.

Parameter	Bleeders (*n* = 18)	Non-Bleeders (*n* = 18)	*p*-Value
% P-selectin^+^ platelets	8.15 ± 4.40	8.26 ± 4.45	0.94
% GP1bα^+^ platelets	97.3 ± 1.5	97.2 ± 2.2	0.95
% CD63^+^ platelets	1.37 ± 3.42	0.65 ± 0.42	0.38
% GPIIb/IIIa^+^ platelets	94.5 ± 23.5	94.4 ± 23.5	1.00
% DHR123^+^ platelets	87.1 ± 9.0	86.1 ± 11.9	0.76
% MitoTracker Green^+^ platelets	19.6 ± 17.7	17.0 ± 19.8	0.68
MFI P-selectin^+^ platelets	1342 ± 334	1369 ± 334	0.81
MFI GP1bα^+^ platelets	672 ± 217	630 ± 124	0.48
MFI CD63^+^ platelets	1448 ± 1084	1564 ± 914	0.73
MFI GPIIb/IIIa^+^ platelets	35,240 ± 9806	34,676 ± 11,088	0.87
MFI DHR123^+^ platelets	742 ± 561	638 ± 386	0.52

CD, cluster of differentiation; DHR123, dihydrorhodamine123; GP, glycoprotein; MFI, mean fluorescence intensity; NSB, non-surgical bleeding.

## Data Availability

Not applicable.
